# Dissecting the shared genetic architecture between endometriosis and polycystic ovary syndrome

**DOI:** 10.3389/fendo.2024.1359236

**Published:** 2024-04-29

**Authors:** Hangjing Tan, Panpan Long, Hongmei Xiao

**Affiliations:** ^1^ Institute of Reproductive & Stem Cell Engineering, Center of Reproductive Health, School of Basic Medical Science, Central South University, Changsha, Hunan, China; ^2^ Center of Genetics, Changsha Jiangwan Maternity Hospital, Changsha, Hunan, China

**Keywords:** endometriosis, polycystic ovary syndrome, genome-wide association study, shared genetic architecture, genetics

## Abstract

**Background:**

Previous study suggested evidence for coexistence and similarities between endometriosis and polycystic ovary syndrome (PCOS), but it is unclear regarding the shared genetic architecture and causality underlying the phenotypic similarities observed for endometriosis and PCOS.

**Methods:**

By leveraging summary statistics from public genome-wide association studies regarding endometriosis (European-based: N=470,866) and PCOS (European-based: N=210,870), we explored the genetic correlation that shared between endometriosis and PCOS using linkage disequilibrium score regression. Shared risk SNPs were derived using PLACO (Pleiotropic analysis under composite null hypothesis) and FUMA (Functional Mapping and Annotation of Genetic Associations). The potential causal association between endometriosis and PCOS was investigated using two-sample Mendelian randomization (MR). Linkage disequilibrium score for the specific expression of genes analysis (LDSC-SEG) were performed for tissue enrichment analysis. The expression profiles of the risk gene in tissues were further examined.

**Results:**

A positive genetic association was observed between endometriosis and PCOS. 12 significant pleiotropic loci shared between endometriosis and PCOS were identified. Genetic associations between endometriosis and PCOS were particularly enriched in uterus, endometrium and fallopian tube. Two-sample MR analysis further indicated a potential causative effect of endometriosis on PCOS, and vice versa. Microarray and RNA-seq verified the expressions of *SYNE1* and *DNM3* were significantly altered in the endometrium of patients with endometriosis or PCOS compared to those of control subjects.

**Conclusion:**

Our study indicates the genetic correlation and shared risk genes between PCOS and endometriosis. These findings provide insights into the potential mechanisms behind their comorbidity and the future development of therapeutics.

## 1 Introduction

Endometriosis is characterized by abnormal presence of endometrial glands and stroma outside the uterus (1, 2), of which symptoms include dysmenorrhea, pelvic pain and infertility. There are approximately 10% of reproductive-aged women diagnosed with this condition. It is a benign disease but with many malignant characteristics, such as estrogen dependence, recurrence and invasiveness (3). The economic burden exceeds US$22 billion in the USA alone (4). Polycystic ovary syndrome (PCOS), with a prevalence rate of approximately 10% (5), is also a disorder affecting reproductive-aged women, which is characterized by enlarged and dysfunctional ovaries, resistance to insulin, excess androgen levels (6, 7). During pregnancy in PCOS patients, their hospitalization rate is twice higher than that of general population, because of pregnancy complications such as diabetes and pre-eclampsia.

The relationship between endometriosis and PCOS is controversial. Some studies suggested evidence for coexistence and similarities between endometriosis and PCOS (8, 9). For example, endometriosis and PCOS have similar symptoms, both of which can lead to infertility and miscarriage (10). Holoch et al. reported high rates of endometriosis in women with PCOS (>70%) (11). In endometrium, endometriosis patients and PCOS patients have similar defects (12, 13). The receptivity of the endometrium to embryos decreases, and the expression of genes related to supporting blastocyst attachment (*MUC1*, *L-Selectin*) in the endometrium is abnormal. The genes that affect the interaction between mother and fetus also express abnormally (14). Adipokines, secreted by white or brown adipose tissues, plays a central role in energy metabolism. The expression level of some adipokines, such as leptin, chemerin, adiponectin, apelin, have similar changes in both endometriosis and PCOS (15). In ovary, CXC chemokines, chemotactic and secreted cytokines, were dysregulated in both PCOS and endometriosis, which may promote the progression of both disease (16). Endometriosis and PCOS also have similar hormone dysregulation. Endometriosis is an estrogen dependent disease accompanied by progesterone resistance. This hormonal imbalance can lead to exacerbation of inflammation, increase of pelvic pain, and reduction of the endometrium receptivity to embryo implantation (17). In PCOS, estrogen activity is also increased and accompanied by progesterone resistance, which promotes endometrial growth (18). Dysregulation of the gut microbiota composition can lead to several diseases in reproductive endocrine system (19). There are similar changes in gut microbiota in patients with endometriosis and PCOS (19). Some studies claimed endometriosis and PCOS are diametric disorders (11). In the ovaries of patients with endometriosis, the rate of follicle recruitment and degeneration accelerates, leading to accelerated depletion of ovarian reserves. In the ovaries of PCOS patients, the speed of follicle transition from primordial reserve to dynamic reserve slows down, and the number of antral follicles increased (11). They supposed that endometriosis and PCOS represent diametric outcomes of variation in hypothalamic-pituitary-gonadal axis development. Endometriosis is mediated by low prenatal and postnatal testosterone, while PCOS is mediated by high prenatal testosterone. Therefore, the association between endometriosis and PCOS needs further exploration.

Both endometriosis and PCOS are heritable disease. It was estimated that about 51% of the variation in endometriosis risk is heritable according to a study including 3,096 female twins in Australian women (20). Genome-wide association studies (GWASs) have identified several independent single-nucleotide polymorphisms (SNPs) for endometriosis. In a Japanese ancestry, rs10965235 in *CDKN2BAS* on chromosome 9p21.3 was identified (21); In a US GWAS from European-ancestry women, rs1519761 on 2q23.3 were identified (22); In an European-ancestry GWAS and from a meta-analysis of European and Japanese ancestry GWAS data, rs7521902 near *WNT4* on 1p36.12, rs13391619 in *GREB1* on 2p25.1, rs4141819 on 2p14, rs7739264 near *ID4* on 6p22.3, rs12700667 on 7p15.2, rs1537377 near *CDKN2B-AS1* (independent of rs10965235) on 9p21.3 and rs10859871 near *VEZT* on 12q22 were identified (23, 24); In an Icelandic GWAS, rs17773813 near KDR on 4q12 and rs519664 in *TTC39B* on 9p22 were identified (25). Sapkota et al. identified five novel genetic loci (*ESR1*, *CYP19A1*, *HSD17B1*, *VEGF* and *GnRH*) which are associated with genes involved in sex steroid regulation and function (26) based on GWAS. These genetic loci may be beneficial for providing diagnostic markers for endometriosis. Based on GWAS, genetic association between endometriosis and other diseases, such as depression, anxiety, and ovarian cancer was also identified. These results implicated endometriosis share a common genetic basis with multiple diseases. Genetic factors has a role in the development of PCOS. It was estimated that the heritability of PCOS is 70% (27). Using linkage and association studies within the population or families, almost 100 susceptibility genes related to PCOS were identified (28). Through GWAS, 11 susceptibility loci mapping to *DENND1A*, *THADA*, *LHCGR*, *FSHR*, *INSR*, *TOX3*, *YAP1*, *RAB5B*, *c9orf3*, *HMGA2*, and *SUMO1P1*/*ZNF217* have been identified in Han Chinese populations, and polymorphism in *CYP11A*, *CYP17*, *CYP19*, *CYP21*, *β-HSD* also result in the phenotypic expression of PCOS (29). However, the shared genetic architecture and causality between endometriosis and PCOS remains largely unknown, and no shared risk loci been reported previously.

**Figure 1:**
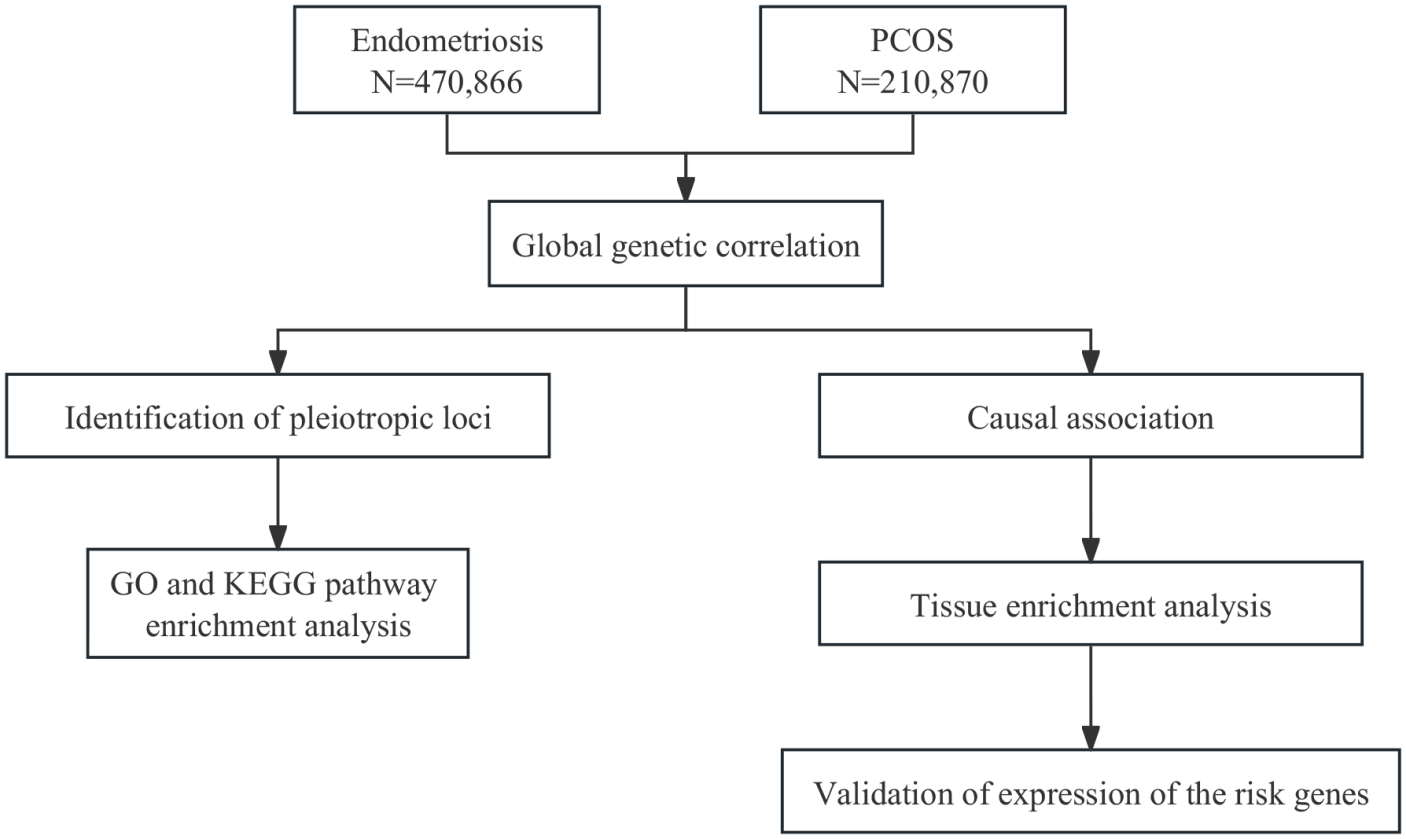
Overall study design. GO, Gene Ontology; KEGG, Kyoto Encyclopedia of Genes and Genomes.

In current study, using large-scale GWAS summary statistics (**Figure 1**), we aimed to investigate the genetic correlation, causal association, and shared risk loci with potential functions between endometriosis and PCOS, and to provide insights into their comorbidity.

## 2 Methods

### 2.1 Datasets

#### 2.1.1 GWAS summary statistics

GWAS summary results for endometriosis were obtained from the GWAS catalog database (https://www.ebi.ac.uk/gwas/, GCST90269970) comprising 21,779 cases and 449,087 controls of European ancestry (30). GWAS summary results for PCOS (consortium definition) were obtained from FinnGen R9 release (https://r9.finngen.fi/) compromising 31,548 cases and 179,322 controls of European ancestry.

#### 2.1.2 GTEx data and Franke lab data

GTEx data is a public data resource of gene expression in 53 nondiseased human primary tissues (31). First of all, we downloaded the GTEx dataset (32), and then chose the lite version of the GTEx V8 expression quantitative trait locus (eQTL) summary data (*P*<1×10^−5^). The Franke lab data set is an aggregation of publicly available microarray gene expression data sets comprising 152 tissues in human, mouse, and rat (33). We downloaded the Franke lab publicly available gene expression data from the DEPICT website (https://data.broadinstitute.org/mpg/depict/depict_download/tissue_expression).

#### 2.1.3 Microarray dataset and bulk-tissue RNA sequencing gene expression data

Two datasets, including GSE7305 and GSE226146, were extracted from Gene Expression Omnibus (GEO) database (https://www.ncbi.nlm.nih.gov/geo). GSE7305 contained ectopic endometrium of eight endometriosis patients and endometrium of eight healthy controls, and the dataset was based on GPL570 (Affymetrix Human Genome U133 Plus 2.0 Array) (34). GSE226146 contained endometrium of three PCOS patients and endometrium of three healthy controls, and the dataset was based on GPL16791 (Illumina HiSeq 2500) (35). All samples were collected during the secretory phase of the menstrual cycle.

### 2.2 Statistical analyses

#### 2.2.1 Global genetic correlation analysis

We used stratified linkage disequilibrium scoring regression (S-LDSC) together with the baseline-LD model to estimate single trait SNP heritabilities (h2SNP) for endometriosis and PCOS. As an extension of S-LDSC, the baseline-LD modeling approach is based on continuous SNP heritability partitioning rather than binary annotation sets. Using the 1000 genomes dataset as a reference panel for the underlying LD structure and limited to HapMap3 SNP (36).

We used bivariate LDSC to estimate the genetic correlation (r_g_) between endometriosis and PCOS. Under the multigene model, the slope of the z-score regression from the LD scores was used to estimate r_g_:


$$\text{E}\left[{\text{z}}_{1\text{j}}{\text{z}}_{2\text{j}}{\text{l}}_{\text{j}}\right]=\frac{\sqrt{{\text{N}}_{1}{\text{N}}_{2}{\text{ρ}}_{\text{g}}}}{\text{M}}{\text{l}}_{\text{j}}+\frac{{\text{N}}_{\text{s}}\text{ρ}}{\sqrt{{\text{N}}_{1}{\text{N}}_{2}}}$$

rg=ρgh12h22

where ${\text{z}}_{1\text{j}}$ and ${\text{z}}_{2\text{j}}$ are the z-scores of SNP j from trait 1 and trait 2, ${\text{r}}_{\text{g}}{\text{ N}}_{1}$ and N_2_ are the sample sizes of trait 1 and trait 2, ρ_g_ is the genetic covariance, l_j_ is the LD score, M is the number of SNPs, N_s_ is the number of overlapping samples, ρ is the phenotypic correlation in the overlapping samples, and ${\text{h}}_{1}^{2}$ and ${\text{h}}_{2}^{2}$ are the heritability of SNPs in trait 1 and trait 2 (37). To assess possible sample overlap between the traits in the pooled GWAS data, we performed LDSC correlation analyses of unconstrained genetic covariate intercepts. LDSC with constrained intercepts was likewise performed as a sensitivity analysis.

#### 2.2.2 Identification of pleiotropic loci

Pleiotropic analysis under composite null hypothesis (PLACO) has been employed to investigate pleiotropic loci associated with complex traits using GWAS association statistics (38). During the process, the squared Z score was calculated for each variant, and SNPs with very high Z^2^ (> 80) values were removed. In addition, given the potential correlation between the two diseases, the correlation matrix of Z was estimated, and its matrix was included in the analysis. Finally, the hypothesis of no pleiotropy was tested using the level-α cross-over-unit test (IUT) method, and the final pleiotropy p-value was determined. Significant pleiotropic variants were defined as single-nucleotide variants with *P*-values less than  5 × 10^−8^. The Functional Mapping and Annotation of Genetic Associations (FUMA) tool was used to delineate potential pleiotropic loci. In the FUMA, SNPs can be annotated based on their physical location to genes, we used FUMA SNP2GENE workflow to define the genomic risk loci and annotate the candidate SNPs. These SNPs were then mapped to genes using ANNOVAR software.

#### 2.2.3 Functional analysis for pleiotropic genes

To gain biological insights for the pleiotropic SNPs, based on PLACO results, we performed multi-marker analysis of genomic annotation (MAGMA) analysis (39) on the genes located in or overlapping with the pleiotropic loci to identify candidate pleiotropic genes. We used the MAGMA analysis in the FUMA platform (40).

The Kyoto Encyclopedia of Genes and Genomes (KEGG) Orthology-Based Annotation System (version 3.0) (41) was used to conduct pathway enrichment analysis and Gene Ontology (GO) was used to better understand the biological mechanisms associated with endometriosis and PCOS.

#### 2.2.4 Mendelian randomization analysis

We applied two-sample bidirectional Mendelian randomization analysis to identify potential causal relationships between endometriosis and PCOS (42). In total, we employed five methods. The main method applied in this study is the inverse variance weighting (IVW). The method pooled the Wald ratio estimates for each SNP, which were obtained by dividing the SNP outcome estimate by the SNP exposure estimate, using the random effects inverse variance method, which weights each ratio according to its standard error and obtains the average chance effect estimate between the two features (43). To increase the reliability and validity of our results, we performed additional analyses using the MR-Egger method, the weighted median method, simple mode, and the weighted mode. MR-Egger regression can be used to detect and correct bias due to horizontal pleiotropy (44). These four methods are not as effective as IVW in detecting true causal effects and are therefore used to complement the findings of IVW.

Valid instrumental variables (IVs) were selected based on three main assumptions: that IVs (1) are associated with exposure; (2) do not depend on confounders; and (3) do not have a direct effect on the outcome (44). Utilizing PLINK, the MR approaches identified independent instrumental SNPs by LD clumping (LD r2< 0.001, within 1000-kb windows). For the MR analysis in this study, we selected SNPs with genome-wide significance (*P* ≤ 5 × 10^−8^) or SNPs with locus-wide significance level (1 × 10^−6^) of the exposure trait as IVs, based on the results of heterogeneity analysis (45). F statistics for each instrument were estimated by F = β^2^/SE^2^ (46). As sensitivity analysis, the heterogeneity Q statistics were calculated. An MR pleiotropy residual sum and outlier (MR-PRESSO) (47) test were performed to detect and correct for horizontal pleiotropy, detecting pleiotropic outliers, and providing outlier-corrected estimates. We then conducted five MR analyses to estimate the causal effect after excluding outlier SNPs.

#### 2.2.5 LDSC-SEG analysis

We performed linkage disequilibrium score for the specific expression of genes analysis (LDSC-SEG) to investigate whether SNP heritability for endometriosis and PCOS was significant for trait tissue correlation inference (48). 53 tissue types are available for GTEx (v.8), and 152 tissues types are available for Franke lab. To further understand the (dis)similarity across traits, we partitioned heritability using stratified LDSC-SEG leveraging genome-wide genetic variants of endometriosis and PCOS. LDSC-SEG was conducted to evaluate the degree of SNP-heritability enrichment of endometriosis and PCOS in specific tissues. And *P* value< 0.05 indicated suggestive significance.

#### 2.2.6 Validation of expressions of the risk gene

The expression profiles of risk genes, which are genes shared SNPs identified by PLACO, were evaluated for the risk gene by the datasets GSE7305 and GSE226146. These two raw datasets were analyzed by GEO2R (https://www.ncbi.nlm.nih.gov/geo/geo2r/), which is an online tool employed to compare two or more samples in different datasets. *P*-value<0.05 and |logFC| >0.5 were defined as differentially expressed genes (DEGs). In GSE7305, we compared DEGs between ectopic endometrium of endometriosis patients and endometrium of healthy controls. In GSE226146, we compared DEGs between endometrium of PCOS patients and endometrium of healthy controls.

## 3 Results

### 3.1 Global genetic correlation between endometriosis and PCOS

We first applied stratified linkage disequilibrium (LD) score regression (S-LDSC) with the baseline-LD model to estimate the SNP-based heritability of endometriosis and PCOS. The observed-scale SNP heritability (unconstrained intercept) was 1.58% for endometriosis, and 2.97% for PCOS. We then used the bivariate LDSC to estimate the genetic correlation between endometriosis and PCOS, genetic correlation (without constrained intercept) between endometriosis and PCOS were identified (r_g_ = 0.56, *P* = 1.81×10^-21^). The calculated values of the intercept of the genetic covariance indicated slight sample overlap (**Supplementary Table S1**). We used a constrained intercept for LDSC without assuming an overall stratification with slightly higher genetic correlations, and the results were still significant (**Supplementary Table S1**).

### 3.2 Identification of shared risk SNPs between endometriosis and PCOS

Based on evidence for significant genetic correlations between endometriosis and PCOS, we performed PLACO to identify risk SNPs underlying the joint phenotypes endometriosis-PCOS. We identified a total of 3,805 single nucleotide variants (SNVs) that exhibited potential polymorphic variants associated with both traits by PLACO (**Supplementary Table S2**). MAGMA analysis yielded 40 significant pleiotropic genes (**Supplementary Tables S3**, **S4**). FUMA further identified 12 independent genomic risk loci that exhibited pleiotropic effects (**Supplementary Table S5**).

### 3.3 Enrichment analysis for identified pleiotropic genes

**Figure 2:**
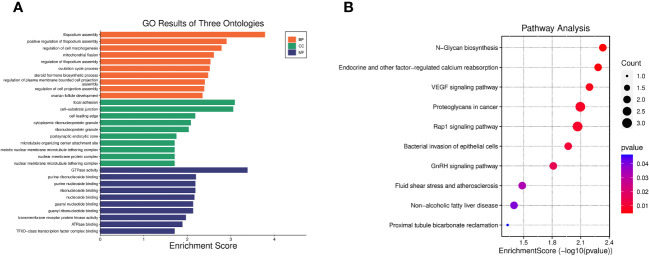
Enrichment analysis of 40 pleiotropic genes. **(A)** Top 10 significant GO results of three ontologies. BP, biological process; CC, cellular component; MF, molecular function. **(B)** Top 10 significant pathways in terms of the KEGG pathway enrichment analyses.

GO enrichment analysis indicated that the 40 pleiotropic genes were significantly enriched in filopodium assembly (*P* = 1.64 × 10^-4^), focal adhesion (*P* = 8.06 × 10^-4^), and GTPase activity (*P* = 4.11 × 10^-4^). The results of the KEGG enrichment analysis demonstrated significant enrichment of genes involved in N-Glycan biosynthesis (*P* = 4.65×10^-3^), endocrine and other factor-regulated calcium reabsorption (*P* = 5.21×10^-3^), and VEGF signaling pathway (*P* = 6.42×10^-3^). **Figure 2** and **Supplementary Table S6** present the GO and KEGG pathways with the top 10 minimum *P*-values.

### 3.4 Mendelian randomization

**Figure 3:**
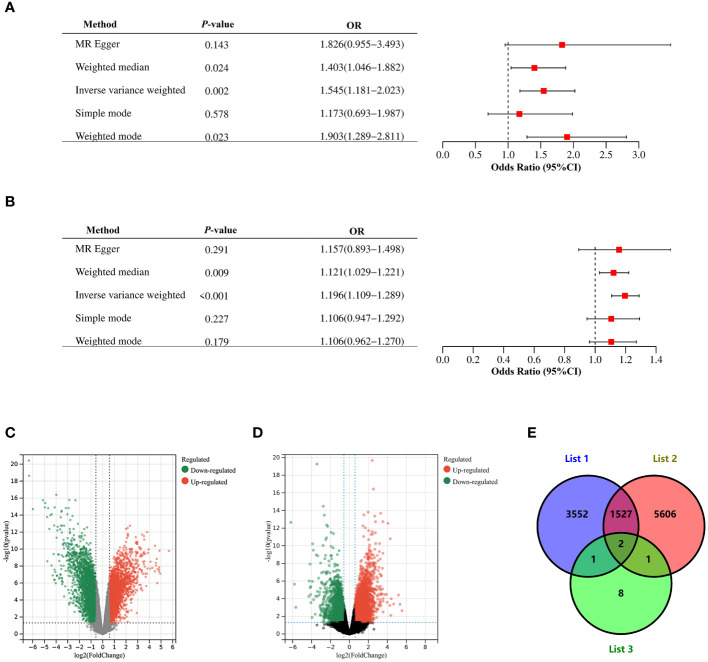
Mendelian randomization and Validation of expressions of the risk gene. **(A)** Causal effect of PCOS on endometriosis. **(B)** Causal effect of endometriosis on PCOS. Estimates and 95% CI were represented with square points and error bars, respectively. In the x-axis, the odds ratio (OR) is the effect of one standard deviation increase in exposure on outcome, at OR = 1, the vertical dashed line represents the reference. **(C)** Volcano plots of DEGs between endometrium of endometriosis patients and endometrium of healthy controls in GSE7305. **(D)** Volcano plots of DEGs between endometrium of PCOS patients and endometrium of healthy controls in GSE226146. Red dots represent significantly up-regulated DEGs, green dots represent significantly down-regulated DEGs, gray dots indicate no significant difference. Genes satisfying the criteria adjust *P*< 0.05 and |logFC| >0.5 were considered significant. Panel **(E)** is the Venn diagram showing common DEGs among List 1, List 2 and List 3. List 1 indicated DEGs in GSE7305, List 2 indicated DEGs in GSE226146, and List 3 indicated 12 pleiotropic genes.

We performed a two-sample bidirectional mendelian randomization analysis to explore whether there is a potential causal relationship between endometriosis and PCOS. The valid IVs were evaluated and screened based on previously mentioned hypothesized criteria. And all SNPs used in the MR analysis were strong instruments (F-statistics >10), indicating a low probability of weak instrumental bias. Various bidirectional mendelian randomization analysis methods were conducted for a more stable results. We first selected SNPs with genome-wide significance (*P* ≤ 5 × 10^−8^) as instrumental variables (IVs). Using IVW, genetic liability to endometriosis (β= 0.18, *P* = 3.09×10^-6^) was significantly associated with an increased risk of PCOS, with no significant evidence of heterogeneity (**Supplementary Table S7**). The estimates remained directionally consistent using weighted median approach (**Figure 3**; **Supplementary Table S7**). MR-PRESSO analysis revealed no outliers in the results. In the reverse MR analysis, using IVW, genetic liability to PCOS (β= 1.16, *P* = 3.71×10^-3^) was significantly associated with an increased risk of endometriosis. However, heterogeneity test showed that Q_pval is less than 0.05 (**Supplementary Table S7**). Therefore, we selected SNPs with locus-wide significance level (1 × 10^−6^) as IVs. Using IVW, genetic liability to PCOS (β=0.44, *P* = 1.52×10^-3^) was significantly associated with an increased risk of endometriosis, with no significant evidence of heterogeneity (**Supplementary Table S7**). The estimates remained directionally consistent using weighted median and weighted model (**Supplementary Table S7**). MR-PRESSO analysis revealed no outliers in the results. Reciprocal causation indicated shared pathogenesis between endometriosis and PCOS.

### 3.5 Tissue-level SNP heritability enrichment

We used LDSC-SEG to assess tissue-level enrichment of SNP heritability for endometriosis and PCOS using genotypic tissue expression (GTEx) and Franke lab data. We identified FDR significant (*P*< 0.05) SNP heritability enrichment for endometriosis across ten tissues (**Supplementary Table S8**). For PCOS, six tissue were significantly enriched (*P*< 0.05) (**Supplementary Table S9**). These results indicated shared genetic basis for endometriosis and PCOS may mainly enriched in uterus, endometrium and fallopian tubes. Therefore, in following validation of expressions of the risk genes, we selected expression profile analysis of endometrium in endometriosis and PCOS.

### 3.6 Validation of expressions of the risk genes

In dataset GSE7305, which contained ectopic endometrium of endometriosis patients and endometrium of healthy controls, 5,082 DEGs were sifted out in total (**Figure 3C**). In dataset GSE226146, which contained endometrium of PCOS patients and endometrium of healthy controls, 7,136 DEGs were sifted out in total (**Figure 3D**). In addition, 12 independent genomic risk loci were identified through PLACO and FUMA. Only two genes, including *SYNE1* and *DNM3*, existed in these three datasets (**Figure 3E**). The expression of *SYNE1* and *DNM3* were significantly decreased in endometrium of both endometriosis and PCOS compared to control subjects. These results indicated *SYNE1* and *DNM3* are shared genes in endometriosis and PCOS, promoting changes of endometrium in both endometriosis and PCOS.

## 4 Discussion

By leveraging large GWAS datasets and tissue specific expression data, our study provided evidence of causality and shared genetic architecture between endometriosis and PCOS.

We found a significant positive genetic correlation between endometriosis and PCOS, which supported the hypothesis that genetic factors play an important role in the comorbidity of these two diseases.

Based on the PLACO, 40 significant pleiotropic genes were identified. GO and KEGG enrichment analysis for these 40 pleiotropic genes indicated biological mechanisms associated with endometriosis and PCOS, and results showed that there are lots of similar pathways involved in endometriosis and PCOS, which may explain the similarities between these two diseases. For example, the receptivity of the endometrium to embryos in both endometriosis and PCOS decreases, and “Regulation of cell morphogenesis” (49–51), “focal adhesion” , “regulation of cell projection assembly” (52) are associated with the development of endometrial receptivity for blastocyst implantation. In addition, in endometriosis and PCOS, estrogen activity is increased and accompanied by progesterone resistance, and “steroid hormone biosynthetic process” (53) are associated with hormone imbalance. There are also differences between these two diseases. For example, “mitochondrial fission” (53) and “GTPase activity” (54, 55) plays opposite roles in two diseases, which may explain the differences between these two diseases, such as the speed of follicle recruitment and degeneration.

In addition to the IVW methods, different statistical methods for Mendelian randomization analysis were used to evaluate the robustness of our results. Since several methods for the causal estimates of endometriosis on PCOS, or estimates of PCOS on endometriosis, were statistically significant, the effect was considered robust in this study. Reciprocal causation indicated shared pathogenesis between endometriosis and PCOS. Our result is also consistent with studies proposed by Yang et al, which indicated that pathogenesis of both endometriosis and PCOS included oxidative stress, inflammation, immune dysregulation (38). Inflammation, resulting from immune dysregulation, is one of the main mechanisms that triggers cell metastasis and invasion, which are critical for development of endometriosis (56). Oxidative stress markers are elevated in endometriosis in comparison to control groups (57). High levels of oxidative stress promote inflammation, angiogenesis and cell proliferation, which may promote the development of endometriosis (57). Oxidative stress and inflammatory markers are significantly correlated with increasing androgen levels by upgrading the activity of enzymes involved in steroidogenesis, which may explain their role in PCOS (58–60).

Functional enrichment for gene expression in multiple tissues and cells was also investigated using the GTEx datasets and Franke lab. We identified ten tissues, with significant SNP heritability enrichment for the endometriosis trait. The enrichment results of the PCOS trait were mainly reflected in six tissues. Additionally, we identified heritability enrichment for endometriosis and PCOS in the uterus, endometrium and fallopian tubes. Consistent with studies proposed by Palomba et al. and Liu et al., there are lots of similarities in endometrium between endometriosis and PCOS (61, 62). For example, compared with healthy controls, pro-inflammatory pathways were enhanced, estrogen receptors were upregulated while progesterone receptors were downregulated, markers of epithelial cells were decreased, in endometrium of both endometriosis and PCOS. However, the similarity between endometriosis and PCOS in the fallopian tubes is currently unclear and needs further research.

Based on the PLACO and FUMA, 12 independent genomic risk loci for endometriosis and PCOS were also identified. In addition, microarray dataset and RNA sequencing dataset indicated the mRNA expressions of *SYNE1* and *DNM3* were downregulated in endometrium of patients with endometriosis or PCOS compared with control groups. *SYNE1* encodes nesprin-1, a nuclear envelope protein, which is critical for binding between cytoskeleton, nuclear envelope and other subcellular compartments (63, 64). Nesprin-1 deficiency leads to abnormal nuclear morphology, cell mobility, and cytoskeleton organization (63, 65). In muscle, SYNE1 is involved in anchoring specialized myonuclei underneath the neuromuscular junctions, and *SYNE1* mutations may cause ataxia (66). *SYNE1* is also downregulated in numerous malignancies, and had notable alterations in 10% of gynecologic malignancies (67). One particular mechanism of SYNE1 in malignancies is based on the fact that nuclei are the stiffest organelles in most cells (68). Since nesprin proteins regulate nuclear morphology and cell mobility, *SYNE1* downregulation in cancer may increase nuclear malleability so that cells can migrate through narrow tissue spaces to invade neighboring tissues. In endometriosis, based on GWAS, meta-analysis, and whole-exome sequencing (WES) analysis on a cohort of 80 endometriosis patients, *SYNE1* is identified as a candidate gene in endometriosis (69). The relationship between *SYNE1* and PCOS, and the mechanisms of *SYNE1* in endometrium of endometriosis or PCOS is currently unclear. Endometriosis is a benign disease but exhibit many malignant features, including invasive, progressive and estrogen-dependent growth (70). PCOS patients may exhibit endometrial hyperplasia and an increased risk of endometrial cancer (71). Therefore, we speculate that downregulation of *SYNE1* may promote migration and invasion of endometrial cells in endometriosis or PCOS by regulation nuclear malleability and morphology. In addition, Sur et al. proposed SYNE1 is involved in sex steroid hormone pathways (26). Endometriosis is a estrogen-dependent and progesterone resistance disease, and the endometrium of women with PCOS often exhibits elevated estrogen activity and progesterone resistance (18). Downregulation of *SYNE1* may also promote development of endometriosis or PCOS by regulating sex steroid hormone, such as estrogen. DNM3 is a tumor suppressor factor. For example, in hepatocellular carcinoma, the downregulation of *DNM3* promoted cell proliferation by increasing cell cycle-associated proteins, including cyclin D1, and the upregulation of *DNM3* induced cell apoptosis and inhibited tumor growth (72). The relationship between *DNM3* and endometriosis or PCOS is currently unknown. Endometriosis exhibit many malignant features, such as invasive and progressive. PCOS patients may exhibit endometrial hyperplasia. Therefore, we suppose that *DNM3* may also promote endometrial cells proliferation and invasion through modulating cell cycle-associated or apoptosis-associated proteins. However, additional research is needed to confirm these possible associations and to elucidate the mechanisms involved.

Our research also has several limitations. First, the vast majority of participants in our study were of European ancestry and not extrapolating to other ancestries. Second, we included only data from autosomes in our study (except MR analyses), because the analysis software does not apply to sex chromosomes. Third, we assessed tissue-specific heritability enrichment based on the top 10% of most specific genes overlooking the effects of other genes. Fourth, our inferred causality is presumptive because it is generated based on GWAS summary statistics. Larger and more robust endometriosis and PCOS GWAS are needed to clarify potential causal relationships. Last, some studies proposed that endometrium and PCOS are diametric disorders, which seems contradictory to our research. We speculated that this may be due to the fact that the two diseases are partially positively correlated, and partially negatively correlated. Future longitudinal studies and experimental work are also necessary to investigate the biological mechanisms behind the observed genetic relationships.

In conclusion, we found significant genetic correlations between endometriosis and PCOS and identified common risk SNPs. The causal relationship between endometriosis and PCOS is reciprocal. *SYNE1* and *DNM3* were potential shared genes between endometriosis and PCOS. These findings may provide insights into the common genetic architecture between endometriosis and PCOS, and contribute to a better understanding of their pathogenesis as well as future therapeutic strategies.

## Data availability statement

The datasets presented in this study can be found in online repositories. The names of the repository/repositories and accession number(s) can be found in the article/**Supplementary Material**.

## Author contributions

HT: Writing – review & editing, Writing – original draft, Visualization, Validation, Supervision, Software, Resources, Project administration, Methodology, Investigation, Formal analysis, Data curation, Conceptualization. PL: Writing – review & editing, Writing – original draft, Validation, Supervision, Software, Project administration, Methodology, Investigation, Formal analysis, Data curation, Conceptualization. HX: Writing – review & editing, Writing – original draft, Software, Project administration, Investigation, Data curation, Conceptualization.
